# A Substantial Role of Agro-Textiles in Agricultural Applications

**DOI:** 10.3389/fpls.2022.895740

**Published:** 2022-06-21

**Authors:** Neha Sharma, Ben Allardyce, Rangam Rajkhowa, Alok Adholeya, Ruchi Agrawal

**Affiliations:** ^1^TERI-Deakin Nanobiotechnology Centre, The Energy and Resources Institute, TERI Gram, Gual Pahari, Gurugram, India; ^2^Institute for Frontier Materials, Deakin University, Waurn Ponds, VIC, Australia

**Keywords:** agro-textile, non-conventional fibers, chemical compositions, retting, application

## Abstract

Agro-textiles have been used in the agriculture sector for thousands of years and are an attractive tool for the protection of crops during their entire lifecycle. Currently, the agro-textile market is dominated by polyolefins or petrochemical-based agro-textiles. However, climate change and an increase in greenhouse gas emissions have raised concern about the future oil-based economy, and petroleum-based agro-textiles have become expensive and less desirable in the modern world. Other products include agro-textiles based on natural fibers which degrade so fast in the environment that their recovery from the field becomes difficult and unattractive even by efficient recycling or combustion, and their lifetime is usually limited to 1 or a maximum of 2 years. Hence, the development of bio-based agro-textiles with a reduced impact on the environment and with extended durability is foreseen to initiate the growth in the bio-based economy. The world is gradually preparing the shift toward a bio-based economy, and research for sustainable bio-based alternatives has already been initiated. This review provides insight into the various agro-textiles used currently in agriculture and the research going on in the area of agro-textiles to offer alternative solutions to the current agro-textile market.

## Introduction

Globally, one of the major issues is food security due to the threat of climatic changes caused by the utilization of non-biodegradable petroleum-based agricultural products. A few reports demonstrated that approximately 10–40% losses occurred in crop production because of the drastic increase in climatic temperature. This created a driving force in the agro-textile sectors to improve the yield and quality of the crop every year (Desai, 2018). Current cultivation practices involve the utilization of herbicides and pesticides to prevent crop losses, however, these interventions are expensive and have a long-lasting ecological impact on soil microflora and the environment (Chowdhury et al., 2017). With a significant increase in environmental awareness and development in technology, considerable attention has been diverted to the utilization of textile fibers in agriculture (Kasirajan and Ngouajio, 2012). The textile sector plays a vital role in the development and circulation of the world economy, so it is considered one of the largest industries among numerous sectors around the world. By utilizing biotechnology, the textile sector brings a revolution to textile processing. Textile processing has led to the synthesis of a huge amount of cellulose fibers that can be used in multiple sectors ranging from biomedical to agriculture (Yusuf, 2021). The cellulose-based agricultural products are environmentally friendly with low manufacturing cost. Subsequently, the other concerns associated with petroleum-based products can be reduced along with greenhouse gas emissions (Janaswamy et al., 2022). Agro-textile products (shade nets, harvest nets, and mulch mats) are capable of supporting agriculture by protecting crops from harsh weather conditions and unwanted pests without impacting the environment as these textile products are biodegradable and non-toxic (Bhavani et al., 2017).

Agro-textiles also help in water conservation, moisture retention, weed suppression, and light reflection (Kumar, 2017). Agro-textiles prevent the soil from drying out and maintain homogeneity thereby increasing the crop yield. Agro-textiles restrict the farmer from the overutilisation of harmful pesticides that have a long-lasting impact on the soil as well as the microflora through the utilization of agro-textile covers such as weed control mats, crop covers, and others. The best-known products are shade nets and thermal screens; their usage can save up to 40% on energy in heating greenhouses. The utilization of shades and nets further improves the quality of fruit, prevents staining, and improves the crop by maintaining the overall uniformity in terms of color. Crop covers sustain an optimal micro-climate which protects plants from adverse weather conditions (Bhavani et al., 2017). Various remarkable properties of agro-textile-based products in agriculture are resistant to ultraviolet radiation and micro-organisms and have high tensile strength and biodegradability over petroleum-based products which make them suitable aspirants to overcome numerous conventional problems of agriculture. Textile covers are capable of retaining 15–60 g/m^2^ of water and some tend to retain around 100–500 g/m^2^ which further facilitates the availability of water under drought conditions (Agarwal, 2013; Kumar, 2017).

### Status of the Global Market

The global market of the agro-textile industry is estimated at US$9.37 billion in 2021 and is expected to reach US$13.04 billion by 2028 with a Compound Annual Growth Rate (CAGR) of 4.7%. A report of the global agro-textile market published in 2021 showed that agro-textiles have a US$ 8.4 billion market that accounts for 6% of the total technical textile market globally. Global demand is directed by developing countries such as China, India, and Brazil (Global Agro Textiles Market Size, Share & Trends Analysis Report by Product by Application by Region, and Segment Forecasts, 2021-2028)^1^. Commercialisation of bio-based agricultural products with increasing global farming standards and technologies is expected to drive the market in the expected period. Asia Pacific is estimated to witness the fastest CAGR from 2021 to 2028. The annual growth rate is credited to the uplifting demand for high-quality agriculture products and increasing awareness of advanced materials by the farmers in the agriculture sector (https://www.researchandmarkets.com/reports/4479690/global-agro-textiles-market-size-share-and). The agro-textile market in India is estimated to reach US$ 14.3 billion in 2025 at a CAGR of 5.5% from 2017 to 2025 (Technavio EMIS Database, 2017). The major vendors manufacturing agro-textiles in the world are given in Table 1.

**Table 1:** Agro-textile manufacturing companies around the globe (Indian Technical Textile Association, dataset taken from global agro-textile market).

**Company name**	**Location**	**Detail of products**
Garware Wall Ropes Ltd.	Maharashtra, India	Fisheries, aquaculture, yarn and threads, coated fabrics, agriculture products
Rishi TechTex Ltd.	Daman & Diu, India	Agriculture/ horticulture field, wind breakers, drying of grapes, etc.
Neo Corp Ltd.	Regional offices: New Delhi, Kolkata, Ahmedabad, India	Agro-textiles, geotextiles, textile products for industrial packaging, etc.
CTM Agro Textile Ltd.	Ahmedabad, India	Shade nets, mulching film, greenhouses, anti-insect nets, vermin beds, crop covers
Fibreweb India Ltd.	Daman & Diu, India	Spun-bonded non-woven polypropylene fabrics
B & V Agro Irrigation Co.	Maharashtra, India.	Shade nets, weed mats, insect mesh, anti-hail nets, monofilament
Admire Fibretex Pvt. Ltd.	Gujarat, India	Crop cover, fruit cover, mulching, weed barrier, garden cover
Fortune Agro Net	Gujarat, India	Shading agro nets, anti-hail nets, greenhouse film, sun shade nets mulching sheets, bird protection nets, etc.
Mechanische Netzfabrik Walter Kremmin GmbH & Co. KG	Germany	Nets and ropes
A-BOS limited	United States of America	Ropes and outdoor products
ACE Geosynthetics Enterprise Co., Ltd.	Taiwan	Geotextiles, geogrids

### Stratification of Fibers Utilized in Agriculture

**Figure 1:**
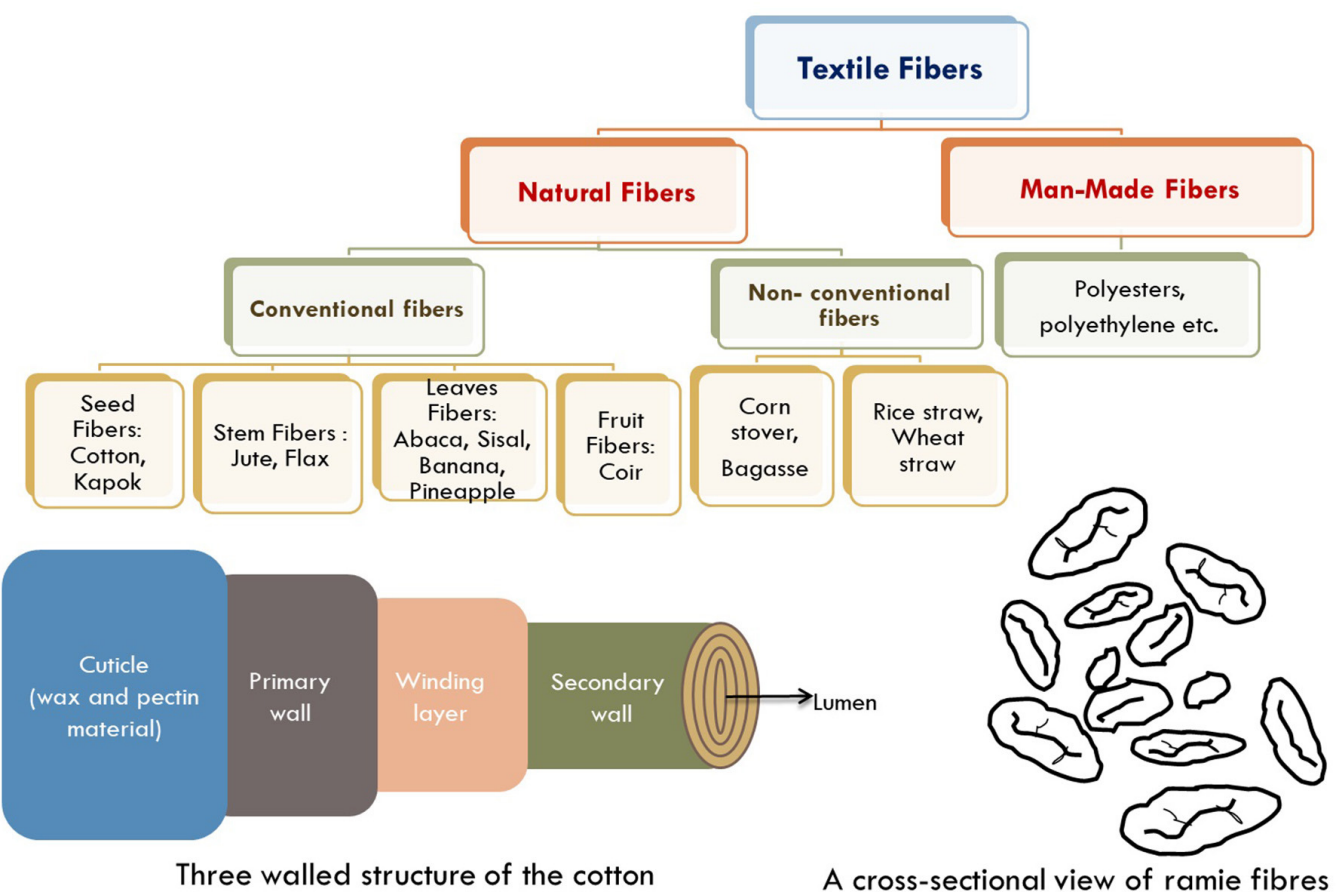
Classification of fibers with three-walled structure of cotton and cross-section view of ramie fibers.

Textile fibers are classified as natural fibers and man-made fibers as depicted in Figure 1. Natural agro-textiles are eco-friendly and are obtained from plant-based or animal-based fibrous materials (Swapan et al., 2016; Scarlat et al., 2017; Restrepo et al., 2019). Plant-based fibers are the dominant form of natural fibers and are obtained from various parts of plants such as seeds, stems, leaves, and fruit. Plant fibers are renewable, biodegradable, and have a long history in human civilization (Blackburn, 2005; Mwaikambo, 2006; Mather and Wardman, 2010). Agro-based fibers are composed of cellulose, hemicellulose, and lignin, and also have silica contents as shown in Table 2.

**Table 2:** Composition and properties of conventional fibers.

**Natural fibers**	**Source**	**Cellulose (%)**	**Lignin (%)**	**Hemicellulose (%)**	**Diameter of fiber (μm)**	**Properties**	**References**
Cotton	Seed	80–90		4–6	12 −45	Hydrophilic, stable in water, resistant to alkali	Sfiligoj et al., 2013; Mathangadeera et al., 2020
Kapok	Seed	64	13	10	30–36	Excellent thermal and insulating properties, high buoyancy	Rijavec, 2008; Chen et al., 2013; Liu et al., 2016
Flax	Stem	60–70	2–3	17	40–80	Crystalline structure contributing to strength	Blackburn, 2005; Mohanty et al., 2005; Dai, 2006; Mather and Wardman, 2010; Goudenhooft et al., 2019
Jute	Stem	61–71	12–13	14–20		Antistatic and highly insulating	Lipp-Symonowicz et al., 2011; Salman, 2020
Hemp	Stem	77	1.7		16–50	Excellent moisture resistance	Mohanty et al., 2005; Nykter, 2006; Tara and Jagannatha, 2011; Musio et al., 2018
Ramie	Stem	93,	0.65	2.5	34	Excellent mechanical properties, resistant to bacteria, mildew, and insects	Mather and Wardman, 2010; Cheng et al., 2020
Kenaf	Stem	45–57	8–13	21.5	38	Tensile strength	Mohanty et al., 2005; Tara and Jagannatha, 2011; Sreenivas et al., 2020
Sisal	Leaf	70	10–20	10–15	200–400	Durable, resistant to saltwater decomposition	Mohanty et al., 2005; Iniya and Nirmalkumar, 2021
Abaca	Leaf	76.6	8.4	14.6	151	Strong, resistant to saltwater decomposition	Blackburn, 2005; Anthony et al., 2020
Henequen	Leaf	77	13	4.8	170	High tenacity	Blackburn, 2005
Coir	Fruit	43.4	45.8	0.25	150–400	Moisture absorption	Ching et al., 2018

### Non-conventional Plant Fibers for Agro-Textiles

Non-conventional plant fibers obtained from corn stover, bagasse, banana, wheat straw, rice straw, pineapple leaves fibers, etc., have potential utilization for crop improvement (Reddy and Yang, 2005). Non-conventional fibers are derived from lignocellulosic agricultural by-products and have potential utilization in textile and paper industries due to their inherent chemical and physical properties. The properties of lignocellulosic-based fiber depend on the chemical composition of the fiber, the source of the fiber, and the extraction procedures (Reddy and Yang, 2005). Generally, the cellulosic waste from crop residues and other agricultural wastes contains 31–60% cellulose, 11–38% pentosans, and 12–28% lignin (Himanish and Sudhir, 2004)^2^.

Due to the orientation of the elevated fibrils, the tensile strength of corn fibers is significantly good but the fibers have low elongation (Li et al., 2012). Various industries are utilizing sugar bagasse as raw material for biofuel production (Sun et al., 2004). The fiber surface of sugarcane bagasse is formed by parallel stripes and is partially covered with residual material whereas, the pith is a more fragile and fragmented structure containing pits, which are small pores connecting neighboring cells on the surface of the walls (Rezende et al., 2011).

Wheat straw is an agricultural residue that is accessible abundantly all over the world and has been used for various applications such as feedstock and energy production (Sain and Panthapulakkal, 2006). According to Liu et al. (2005), wheat straw has a unique physical structure that makes it a suitable candidate for structural composites. Bamboo fibers have the potential for the composite industry and possess numerous excellent properties (Liu and Hu, 2008; Khalil et al., 2012). The crystallinity and structural characteristics of various non-conventional fibers are depicted in Table 3.

**Table 3:** Characteristics of non-conventional fibers derived from agro-waste.

**Non-conventional fibers**	**Length (mm)**	**Width (μm)**	**Crystallinity (%)**	**Properties**	**References**
Corn stover	0.5–1.5	10–20	48–50	High tensile strength	Reddy and Yang, 2005
Sugarcane bagasse	0.8–2.8	10–34	–	High tensile strength	Reddy and Yang, 2005
Banana leaves	0.9–4.0	80–250	45	Light weight, strong moisture absorption	Subagyo and Chafidz, 2020
Wheat straw	1.32	12.9	55–65	Excellent strength and stiffness	Chen and Liu, 2014
Rice straw	0.92	8.1	40	Significant strength	Chen and Liu, 2014
Pineapple leaves	3–8	7–18	44–60	Higher bending properties	Franck, 2005
Bamboo fibers	2.0	6–12	–	Excellent thermal conductivity, high tenacity	Nayak and Mishra, 2016

### Approaches for Extraction of Fibers From Plant Sources

**Figure 2:**
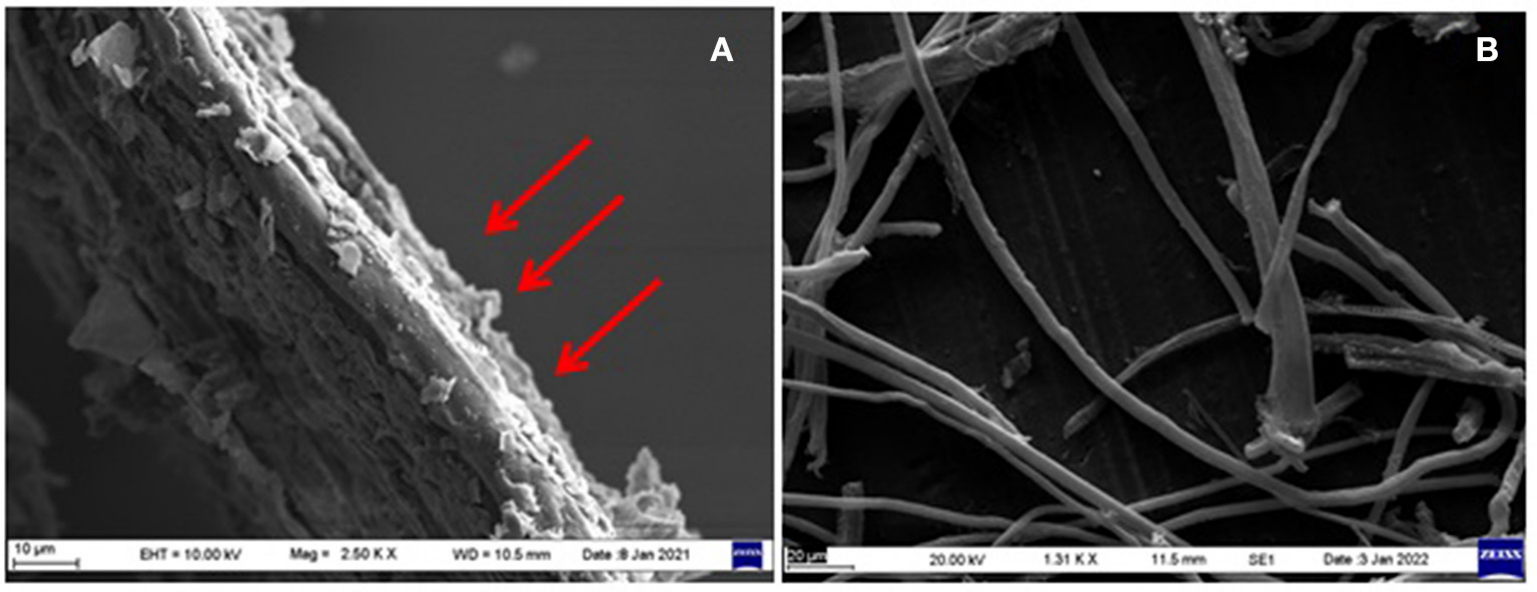
Scanning electron micrograph of: **(A)** untreated rice straw fibers, and **(B)** chemically treated rice straw fibers.

Natural fibers in their native form before any treatment have waxes and other substances on their surfaces that form a thick outer layer to protect the cellulose inside the fibers. The presence of encrusting substances causes the fibers to have an irregular appearance, as shown in Figure 2. Natural fibers are extracted through biological, chemical retting, mechanical, and enzymatic methods. Retting is a process used to extract bast fibers by immersing them in water. Biological retting involves bacteria and fungi to remove lignin, pectin, and other unwanted substances from the fibers and facilitate the accessibility of cellulose (Reddy and Yang, 2005). Water retting is effectively performed using two bacteria (*Bacillus* and *Clostridium species*) whereas dew retting involves fungi (*Rhizomucor pusillus* and *Fusarium lateritium*) for effective removal of a non-cellulosic substance from the fibers (Reddy and Yang, 2005).

**Figure 3:**
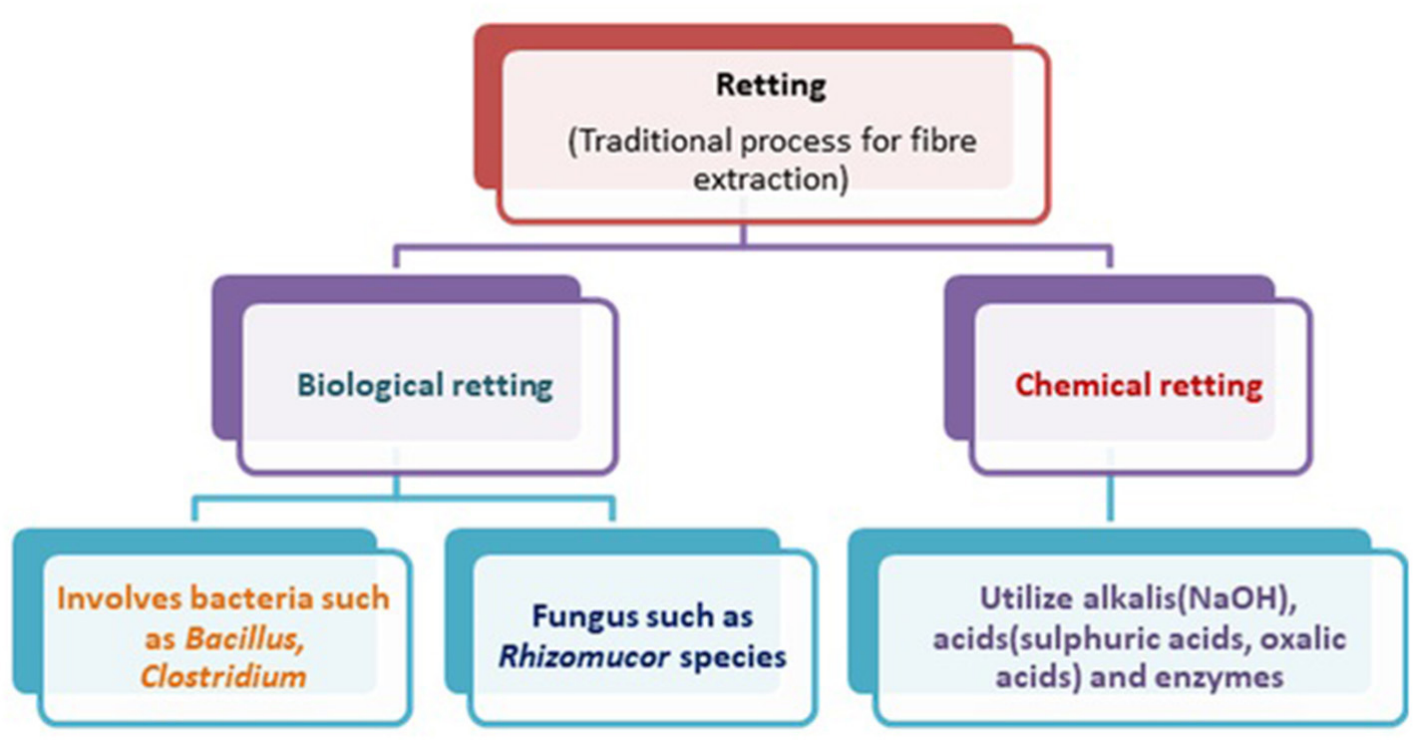
Schematic representation of retting process.

The chemical process involves the utilization of alkalis or mild acids for the extraction of fibers. In the case of alkali retting, sodium hydroxide is commonly used while in acid retting, sulfuric acid and oxalic acid are combined with detergent for efficient extraction of specific fibers. Various factors influence the quality of fibers, *viz.*, duration of treatment, temperature, and concentration of chemicals (Reddy and Yang, 2005). Further, the steam explosion method is a greener approach for the extraction of fibers. It involves the disruption of the cell wall with the help of steam which tends to enhance the availability of cellulose (Gollapalli et al., 2002). The extraction process is listed in Figure 3.

**Figure 4:**
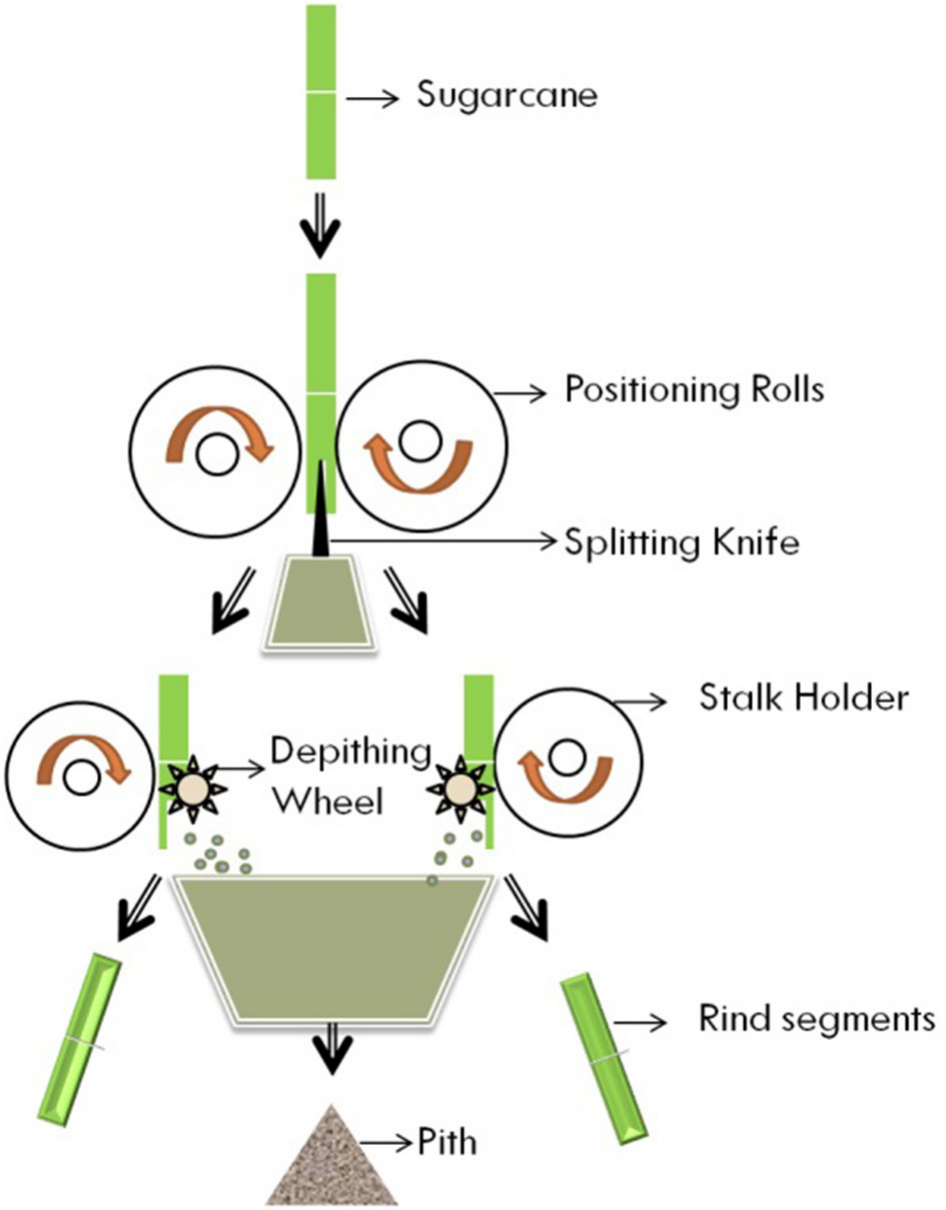
Pictorial of Tilby process of sugarcane extraction.

The enzymatic extraction process involves a combination of enzymes such as pectinases, hemicellulases, and cellulases with pre- or post-chemical treatment. Reddy et al. investigated whether multi-enzyme complexes (10–15 enzymes) efficiently improve the quality of fibers (Reddy and Yang, 2005). Fibers are extracted from the stem through mechanical processing which involves decorticating machines (involving compressive force for stripping the barks, rinds, wood, and plant stalks for further processing). After decortication, fibers are chemically treated to remove lignin, pectin, etc. Another well-known example of cane separation is the Tilby process which separates sugarcane into three materials that are the outer skin, rind, and sugar-containing pith as shown in Figure 4. The outer skin of sugarcane is rich in wax that can be separated for pharmaceutical use. The separated rind can be used for the manufacture of paper, panels, and boards. The third part is pith which is used in the manufacturing of fibreboards, absorbents, and fillers (Tilley patent no. 3567511).

### Application of Agro-Textile Products in the Agriculture Sector

Natural and synthetic fibers are used in various applications related to agricultural crop practices (shade net, plant net, mulch net, insect net, bird net, etc.), horticulture, and floriculture (Basu, 2011). Different agro-textile products are composed of either natural or synthetic fibers as shown in Table 4.

**Table 4:** Applications of agro-textile products in agriculture.

**Agro-textile product**	**Fiber type**	**Textile type**	**Stability to UV radiation**	**Use in agriculture**	**References**
Sunscreen	Polyethylene	Woven	Stable	To protect the crop from UV radiation	Subramaniam, 2009; Agarwal, 2013
Anti-bird net	Polypropylene, polyethylene	Knitted	Stable	Protect crop from birds	Chowdhury et al., 2017
Root ball net	Cotton, polyester, cotton-polyester blend	Woven	^*^NA	Helps to maintain soil on the surface of roots and protect root balls during transportation	Agarwal, 2013
Mulch mat	Wool, polypropylene	Non-woven	Stable	Prevents the growth of unwanted weed, wool mulch mats preserve soil moisture	Agarwal, 2013
Soil protection fabric	Jute, polypropylene	Woven	^*^NA	Prevent soil erosion	Desai, 2018
Harvesting net	Cotton, nylon	Knitted	Stable	Net helps to protect fruits from mold and make harvesting easier	Subramaniam, 2009
Crop cover	Polypropylene	Plastic sheets	Stable	Protect crop from adverse climatic conditions	Desai, 2018
Anti-hail net	Polyethylene monofilament	Knitted	Stable	Guard vines from hail	Chowdhury et al., 2017
Sleeve/bags for nursery	Jute	Woven		For the growth of seedlings	Ghosh et al., 2016

* *NA means the information was not available*.

### Agro-Textile-Based Nets and Their Significance

A shade net is mainly made up of polypropylene and polyethylene which are treated with UV-resistant agents during fiber manufacturing to provide enhanced resistance to UV degradation. The polyethylene polymer is a relatively low-melting material (137°C). Bird protection nets are developed from polypropylene or high-density polyethylene (HDPE) monofilament yarn, these yarns are ultraviolet (UV)-stabilized and knitted into a durable mesh fabric (Preston, 2016). Sunscreen, insect meshes, weed control fabric, and greenhouse covers are typically made of UV-resistant polyethylene fibers.

Various agro-textile products are commonly implemented in the agricultural field such as bird protection nets which offer the passive protection of seeds, crops, and fruits from damage caused by birds. The open mesh net fabrics not only prevent the crop from birds but also provide excellent air circulation which facilitates the optimum growth of the plant; plant nets are composed of polyolefin fibers which are mainly used for tomato crops. The purpose of the plant net is to keep the fruits away from the damp soil which eventually decays the crop. The plant net allows the fruit to grow vertically; monofil nets are designed to protect young branches and flowers against blustery weather and also prevent sand and wind erosion. The nets are set at a right angle to the wind to protect plants against the harmful effects of adverse weather conditions; root ball nets are designed in such a way that when transplanted, roots can protrude through them. They are developed for safe and speedy growth of young plants as the root system remains intact through the net; insect protection nets are made up of polyethylene monofilament meshes to preclude insects from the greenhouses; weed control fabrics halt the growth of unwanted weeds in an environmentally friendly manner and also allow air, water, and fertilizer to sieve through the fabric for plant growth; and fruit covers are made up of non-woven fabric which promotes better growth and enhances the harvest. They tend to protect vegetables, fruits, and plants against snow, rain, frost, and heat (Preston, 2016).

### Coir-Based Products and Their Effect on Plants

A variety of coir-based products are widely used in agriculture such as erosion control blankets, basket liners, bio-rolls, grow sticks, etc. An erosion control blanket is composed of a woven coir mat that protects seeds or seedlings from wind and rain and further facilitates growth, it also protects soil from erosion and mulching action. The basket liners of the coir provide better aeration for growth as air can flow more effectively through the holes of the coir pad. They also help in the vigorous growth of roots.

Bio-rolls are composed of non-woven coir filled with a coir pith composite which facilitates rapid root growth. A growth stick consists of a wooden pole wrapped with coir fibers which provides support to the plant or a creeper. Other natural fibers such as hemp and sisal are used to develop baler twines which are made up of two or three threads twisted together. Baler twines are used for crop wrapping (tomato, grape yards) (Bhavani et al., 2017).

### Agro-Textile-Based Delivery of Fertilizers for Enhancing Plant Yield

**Figure 5:**
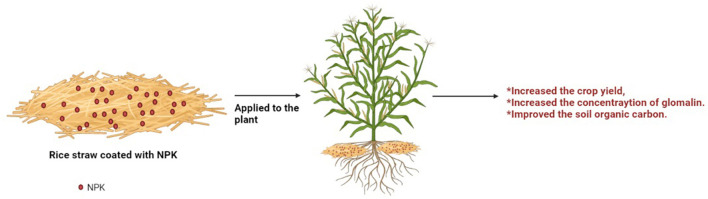
Schematic representation of utilization of rice straw-coated NPK.

Agro-textiles are used as a carrier material where fertilizers are coated through absorption or adsorption onto the non-conventional fibers that further lead to improving crop yield. Nie et al. demonstrated that the combination of fertilizer (nitrogen, phosphorous, potassium) with rice straw tends to elevate the concentration of soil extractable glomalin and total glomalin. The glomalin is the glycoprotein secreted by arbuscular mycorrhizal fungi (AMF) which maintain the stability of soil aggregation. AMF are root symbiotic fungi that play a crucial role in maintaining the soil environment by extending the root system into the soil. Glomalin was correlated with soil carbon and nitrogen, linked with an oligosaccharide, and sequestered toxic elements in the soil. The total glomalin concentration of NPKs (nitrogen, phosphorous, and potassium) was increased to 5.67% when compared with the control plot. Along with that, it also enhanced soil organic carbon and total nitrogen content as illustrated in Figure 5 (Nie et al., 2007).

Geng et al. studied whether coated fertilizers (polymer-sulfur-coated urea) can improve crop productivity, soil organic matter, and also nitrogen use efficiency (NUE). Three field implementations of fertilizer (urea coated with sulfur and thin polymer) with the rate of 180 kg ha^−1^, 126 kg ha^−1^, and 90 kg ha^−1^ on rice (*Oryza sativa L*.) and oilseed rape (*Brassica napus L.)*. The yield of rice and oilseed rape was improved by 6.1–8.2% and 6.3–15.5%; furthermore, the nitrogen use efficiency was enhanced by 15.4–38.4% (Geng et al., 2015). Environmentally friendly fertilizers have numerous merits as they cause less leaching of nutrients, maintain soil fertility, and further reduce the labor cost as compared to conventional fertilizers (Chen et al., 2018).

### Soil Strengthening Using Agro-Textile Fibers

In a study, the strengthening of cohesive soil was investigated through kerosene-coated coir fiber. Kerosene served as a coating agent that tended to lower the water absorption capacity of coir by up to 170%. The purpose of kerosene coating was to prevent the coir fiber from moisture-induced degradation which further imparts strength and stress-strain response of high-plasticity clay up to 52% (Lestelle et al., 2020). Brahmachary et al. showed the improvement of soil with the utilization of natural bamboo fibers enhanced the strength and stiffness of soil. The quantity of bamboo fiber had a direct relationship with the “California bearing ratio” value of soil (a penetration test that determines the force per unit area), which was considerably increased at 1.2% bamboo fiber dosage (Brahmachary and Rokonuzzaman, 2018).

Kanayama et al. cited that bamboo fibers combined with soil at the ratio of 0, 1, 3, and 5% showed the compression stress of 115, 108, 130, and 152 kN/m^2^ respectively. This suggests that lower stiffness has a direct correlation with the strength of the soil (Kanayama and Kawamura, 2019).

### Role of Agro-Textiles in Crop Improvement

Several scientists have proved the influence of jute agro-textiles in the improvement of broccoli productivity, 800 g/m^2^ of jute agro-textile showed a significant result with an average weight of broccoli of 1.2 kg and length of 29.5 cm. The yield was around 4.44 tons/ha and the moisture-holding capacity was 49.05%. The jute agro-textile is composed of a natural jute bast fiber product which is eco-friendly and biodegradable and facilitates plant growth by providing essential plant nutrients through lignin decomposition (Sarkar et al., 2018).

Another study investigated the effect of non-woven ramie fiber film on the root zone environment of rice seedlings. The film was used as a pad on the bottom surface of the seedling tray which tended to enhance the oxygen supply that promoted root respiration; therefore, it had a direct impact on the growth of seedlings. The result of the study showed a significantly higher concentration of soil inorganic nitrogen and decreased organic matter in soil which led to enhanced growth and development of rice seedlings (Zhou et al., 2020).

A group of scientists used non-woven agro-textiles of 10 g m^−2^ and 17 g m^−2^ mass per unit area to protect radish seeds from spit germination and low temperature. Both agro-textile covers enhanced the temperature during the daytime in contrast to the uncovered control plot and tended to improve the germination by around 19% and reduced the germination time (Rekika et al., 2008).

### Other Applications

Jiang et al. (2020) investigated multifunctional TENG (triboelectric nanogenerator) yarn integrated with agro-textiles for various applications. TENG yarn has excellent mechanical and stretchability properties which make it suitable to be woven into a net and incorporated into agro-textiles. TENG is capable of harvesting energy from irregular and low-frequency mechanical energy in the environment that may offer an innovative pattern to take advantage of the rainy phenomenon. Hence, in the meantime, TENG protects crop plantation and the yield of agricultural products; raindrop energy can also be harvested (Jiang et al., 2020).

### Next-Generation of Agro-Textiles: Innovative Production of Mulches and Biodegradable Pots With a Novel Outlook

Plastic-based mulching film utilization in horticulture extensively caused a serious environmental impact due to non-biodegradability. The plastic film waste causes environmental pollution, so to mitigate such problems, biodegradable and renewable materials were used for soil mulching. Various biodegradable materials are natural polymers including starch, cellulose, and chitosan which were efficiently designed to retain their mechanical and physical properties during their implementation until the end of their life span. These materials were directly introduced into the soil as they have been degraded by soil microflora which converts them into carbon dioxide or methane and water (Santagata et al., 2014).

**Figure 6:**
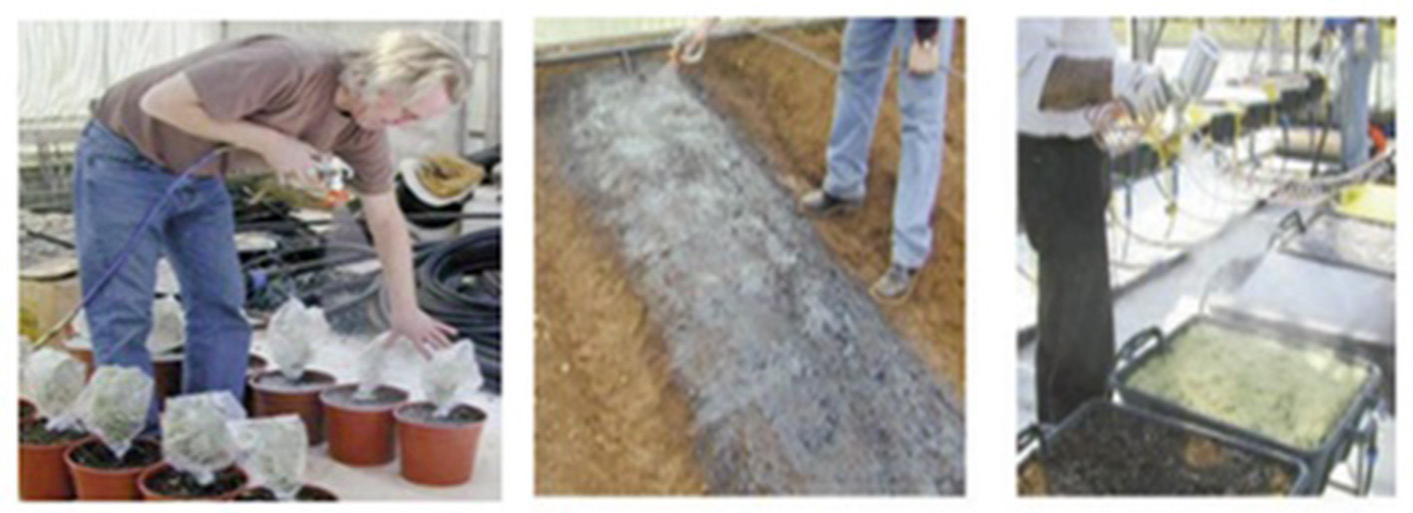
Representation of mulching of soil with biodegradable material through spraying techniques in a cultivated pot (Source: Vox et al., 2013).

The biodegradable extruded mulching films were formed through the thermo-plasticising process (polymer was softened when heated and hardened when cooled down). The spray of mulch solution (water-based natural polysaccharide solution) was released on the field, thus covering the cultivated soil with a protective thin geo-membrane (Figure 6). Another advantage of spraying technology is to avoid the use of film layer machines, which are necessary to apply and remove plastic films (Vox et al., 2013; Malinconico, 2019). A report by Malinconico et al. showed the synthesis of mulching film using cellulose fibers, carbon black, fine bran of wheat, and powdered seaweeds that not only improved the mulching function but also enhanced the tensile strength of the coating material (Malinconico, 2016).

**Figure 7:**
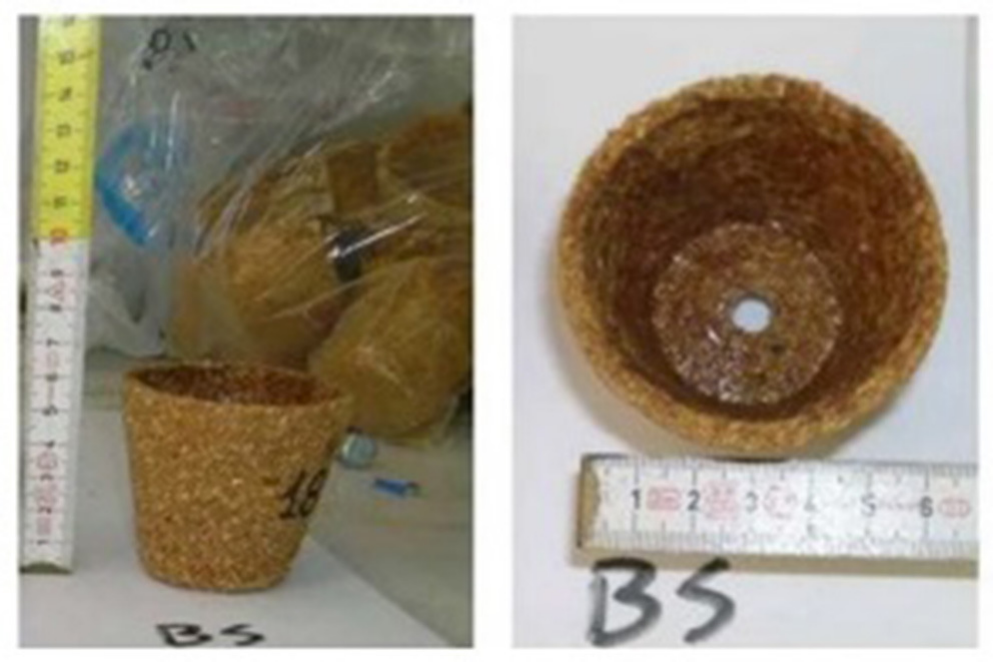
Representation of the biodegradable pot (Source: Sartore et al., 2016).

Another popular utilized practice in horticulture is transplantation where the seedlings are transferred into the soil or into a large container. Commonly, farmers use pots or cell trays composed of fossil raw materials such as polystyrene, polyethylene, and polypropylene that resist proper root growth as roots tend to circulate over the root ball along during transplanting, but the roots can be damaged. An effective alternative to petroleum-based thermoplastic pots may be biodegradable pots that are engineered in such a way that water, air, and roots will penetrate the walls of the pot that ensure healthy root growth (Figure 7). The biodegradable pots can be planted directly into soil, ensuring no pot disposal, reducing cost, labor, and environmental pollution. The mechanism of biodegradation of pots involves the conversion of pots into biomass and inorganic products (e.g., carbon dioxide and water) (Juanga-Labayen and Yuan, 2021).

### Recycling of Agro-Textiles

The current best practice involves the production of value-added products from waste material toward the fulfillment of a sustainable approach which is the part of the circular economy. A circular economy revolves around utilizing textile waste to develop new products. Numerous key elements associated with the circular economy, *viz.*, the utilization of renewable and reusable resources as a raw material, remanufacture resources to enhance the life span of the product, and use the waste resource to recycle or reuse the waste. To achieve this, materials that are meant for landfills or incineration can be recycled into new products/ materials with better quality and higher environmental value (Todor et al., 2019).

A report by Bhatt et al. developed household utility items like lampshades, doormats, wind chimes, coasters, and hanging baskets from defective and damaged textile material used in the university research farms. This fulfills the goal of maintaining the aspect of sustainability with minimal environmental harm and also reduces waste generation. Therefore, waste agro-textiles can be used to generate value-added products that cut down textile waste that is supposed to be disposed of (Bhatt et al., 2019).

### Major Agro-Textile Projects

The seventh framework program includes the development of new agro-textiles with a tailored biodegradability from renewable resources and BIOAGROTEX, Belgium. The BIOAGROTEX project aims to develop a completely bio-based agro-textile with controlled durability as an alternative to existing PP-based agro-textiles or natural fiber-based agro-textiles with a very short lifespan. Manufacturing of biopolymer formulations using various fiber extrusion techniques include tape or monofilament, staple fiber, and multifilament extrusion on laboratory, pilot, and industrial scales including a range of further industrial processing trials such as knitting, weaving, and needle felt production. Two families of biopolymers are evaluated. The use of bio-polyesters as melt-processable polymers, with a focus on PLA, and the use of starch-based formulations. Natural fibers from recycled or from low-value agricultural fractions, and property optimisation by (enzymatic) pre-treatment to optimize yield and properties are developed. Bio-resins (furan-based) for refining NF-based products, extending the self-life without affecting the mechanical properties, processing experimental fibers into non-woven structures and finishing them on a pilot scale, and further scaling to fully integrated industrial processes are developed. Both pathways are supported by laboratory-scale biodegradation tests and detailed chemical analysis of the degradation routes along with the evaluation of the ecological impact and the possible ecotoxicity tests. A set of optimized biopolymer resins or thermoplastics has been defined and can be processed with existing machines to provide excellent processability and properties. Based on these results, different types of agro-textiles can be defined and used directly in the field. The first commercial achievements have already been achieved and hundreds of thousands of m^2^ of specialized agro-textiles are already on sale based on the current development on the market (https://cordis.europa.eu/project/id/213501/reporting).

## Conclusion and Future Perspectives

Agro-textiles provide potential applications in agriculture fields where agro-textile covers help crops to germinate and grow faster. Moreover, it has an overall impact on the plant morphology such as higher plant heights and leaf areas. A variety of fibers from agro-textiles tends to help by improving soil conditions, and also provide organic carbon to the soil when degraded. Next-generation agro-textiles seem to have an inclination toward eco-friendly and biodegradable material drawn from agriculture or farms. Hence, lignocellulosic-based agro-textiles may prove to be an economic and environmentally friendly alternative and may be a boon for agriculture.

## Author Contributions

NS contributed to the writing, editing, and revisions of this manuscript. AA and RA are chiefly responsible for executing the idea, managing funding, and article processing for the final submission. RR and BA critically reviewed the article and provided valuable input for multiple sections. All authors contributed to the article and approved the submitted version.

## Conflict of Interest

The authors declare that the research was conducted in the absence of any commercial or financial relationships that could be construed as a potential conflict of interest.

## Publisher's Note

All claims expressed in this article are solely those of the authors and do not necessarily represent those of their affiliated organizations, or those of the publisher, the editors and the reviewers. Any product that may be evaluated in this article, or claim that may be made by its manufacturer, is not guaranteed or endorsed by the publisher.

## Footnotes

^1^Indian Technical Textile Association. Available online at: http://ittaindia.org/?q=Agro-textile-service-view.

^2^ http://www.agrotech.sasmira.org/about_coe.html#preamble
